# Differential expression of miRNAs in colon cancer between African and Caucasian Americans: Implications for cancer racial health disparities

**DOI:** 10.3892/ijo.2014.2469

**Published:** 2014-05-27

**Authors:** ELLEN LI, PING JI, NENGTAI OUYANG, YUANHAO ZHANG, XIN YU WANG, DEBORAH C. RUBIN, NICHOLAS O. DAVIDSON, ROBERTO BERGAMASCHI, KENNETH R. SHROYER, STEPHANIE BURKE, WEI ZHU, JENNIE L. WILLIAMS

**Affiliations:** 1 Division of Gastroenterology, Stony Brook University, Stony Brook, NY 11794-8160, USA; 2 Division of Cancer Prevention, Stony Brook University, Stony Brook, NY 11794-8160, USA; 3 Division of Colon and Rectal Surgery, Stony Brook University, Stony Brook, NY 11794-8160, USA; 4 Department of Pathology, Stony Brook University, Stony Brook, NY 11794-8160, USA; 5 Department of Applied Mathematics and Statistics, Stony Brook University, Stony Brook, NY 11794-8160, USA; 6 Department of Preventive Medicine, Stony Brook University, Stony Brook, NY 11794-8160, USA; 7 Division of Gastroenterology, Washington University School of Medicine, St. Louis, MO 63110, USA

**Keywords:** African American, Caucasian American, microRNA, colon cancer, quantitative reverse transcriptase-polymerase chain reaction, repeated measures analysis of variance, racial health disparity

## Abstract

Colorectal cancer (CRC) incidence and mortality are higher in African Americans (AAs) than in Caucasian Americans (CAs) and microRNAs (miRNAs) have been found to be dysregulated in colonic and other neoplasias. The aim of this exploratory study was to identify candidate miRNAs that could contribute to potential biological differences between AA and CA colon cancers. Total RNA was isolated from tumor and paired adjacent normal colon tissue from 30 AA and 31 CA colon cancer patients archived at Stony Brook University (SBU) and Washington University (WU)-St. Louis Medical Center. miRNA profiles were determined by probing human genome-wide miRNA arrays with RNA isolated from each sample. Using repeated measures analysis of variance (RANOVA), miRNAs were selected that exhibited significant (p<0.05) interactions between race and tumor or significant (fold change >1.5, p<0.05) main effects of race and/or tumor. Quantitative polymerase chain reaction (q-PCR) was used to confirm miRNAs identified by microarray analysis. Candidate miRNA targets were analyzed using immunohistochemistry. RANOVA results indicated that miR-182, miR152, miR-204, miR-222 and miR-202 exhibited significant race and tumor main effects. Of these miRNAs, q-PCR analysis confirmed that miR-182 was upregulated in AA vs. CA tumors and exhibited significant race:tumor interaction. Immunohistochemical analysis revealed that the levels of FOXO1 and FOXO3A, two potential miR-182 targets, are reduced in AA tumors. miRNAs may play a role in the differences between AA and CA colon cancer. Specifically, differences in miRNA expression levels of miR-182 may contribute to decreased survival in AA colon cancer patients.

## Introduction

In the United States the incidence and mortality rates from colorectal cancer (CRC) are higher in African Americans (AAs) than for all other racial and ethnic groups (1). Socio-economic differences may contribute to delayed detection of cancers. However, a relatively recent study reported that despite receiving equal treatment and after controlling for known prognostic factors, AAs with high-grade tumors were three times more likely to die as a result of CRC than Caucasian Americans (CAs) with high-grade tumors (2). The most recent population-based study shows a 30–50% higher rate of disease-specific mortality after diagnosis among AAs than in CAs (3–8) and this disparity between the 2 groups has greatly widened in recent years. This has occurred despite an increase of CRC screening for both groups. Because one would not expect incidence rates to increase with increased screening and/or detection, it is highly unlikely that socioeconomic status and dietary factors, factors normally attributed to this phenomenon, are the only determinants of racial cancer disparity. Additionally, AAs are diagnosed with CRC at a younger age than Caucasian Americans (CAs) (9). The observation that distinctive mutations in mismatch repair genes hMLH1 and hMSH2 (10), high microsatellite instability (MSI-H), and unique polymorphisms in the *p53* tumor suppressor gene (11) are more prevalent in AA colon cancers than in CA colon cancers, lend support to the concept that biological differences between AA and CA tumors may contribute to increased mortality in AA patients.

miRNAs are frequently dysregulated in cancer and have shown promise as tissue-based markers for cancer classification and prognostication (12,13). Initially synthesized as long primary transcripts, miRNA are processed to small (17–22 nt) regulatory RNAs through a series of steps. miRNAs control gene expression via specific sites at the 3′-UTR of their target mRNAs by accelerating mRNA degradation and/or by repression of translation. A number of studies have reported differential expression of miRNAs in paired colon cancer tumor and adjacent normal colon samples (14–21). miRNAs have also been shown to act either as oncogenes [e.g., miR-155, miR-17-5p and miR-21 (22,23)] or as tumor suppressors [e.g., miR-15a, miR-16-1 and let-7 (24)]. In addition, other studies have indicated that miRNAs are involved in tumor migration and invasion (25). Together, these findings support the premise that the dysregulation of miRNAs can lead to the development of cancer.

In this investigation, microarray analysis and qRT-PCR technologies are used to determine whether miRNA levels are disproportionately expressed in the tumors of AA CRC patients as compared to those of CA CRC patients. In this study, we report the identification of miR-182 as a potential candidate that may contribute to increased colon cancer mortality in AA compared to CA patients.

## Materials and methods

### Ethics statement

This study was approved by the Washington University School (WU) of Medicine-St. Louis and Stony Brook University (SBU) Institutional Review Boards. Tissues were banked at the SBU (http://www.stonybrookmedicalcenter.org/pathology/biobank) and WU (http://www.siteman.wustl.edu/ContentPage.aspx?id=243) human bio-specimen bio-banks. The samples and clinical metadata were de-identified, assigned a patient code and a sample code prior to release to the researchers and qualified for a waiver of consent per 45CFR46.116.d.

### Demographics of colon cancer subjects

The available clinical metadata for the WU samples were limited to age at the time of surgical resection of the tumor, gender (male vs. female) and race (AA or CA). Paired tumor and normal colon RNA samples were prepared from 30 AA and 31 CA subjects. The average age of the CA (63.4±3.5) and AA (61.3±3.6) subjects were not significantly different. Similarly, the gender distribution was not significantly different for AA (15 male, 14 female and 1 unspecified) and CA (17 male and 14 female) patients.

### Extraction of RNA from SBU and WU colon cancer tumor and adjacent normal colon tissue samples

For the SBU samples, sections of 30 pairs of formalin-fixed paraffin-embedded (FFPE) tumor and adjacent normal colon tissues were obtained through the Stony Brook Research Histology Core Lab from 15 CA and 15 AA colon cancer patients who underwent colon cancer surgery at Stony Brook University Medical Center. All SBU tissue sections were reviewed by a surgical pathologist (K.R.S.). Tumor sections selected contained a minimum of 70% neoplastic cells, ensuring that the majority of tissue extracted was of neoplastic origin. The control samples were adjacent normal colon tissue removed during colon resection. Total RNA was isolated from three 10-μm thick tissue sections using a miRNeasy FFPE kit (Qiagen, Valencia, CA). Briefly, tumor sections, as determined morphologically by H&E staining, were removed from slides by scraping with a scalpel blade. Paraffin was removed from the sample using xylene and an ethanol wash. Cells were lysed by digestion with proteinase K at 56°C for 15 min followed by additional incubation at 80°C for 15 min. The supernatant was treated with DNase, followed by the addition of Cell Lysis Buffer and ethanol. Total RNA, including miRNA, was bound to an RNeasy MinElute column (Qiagen). The column was washed twice with RPE buffer (Qiagen) and total RNA eluted with RNase-free water.

For the WU sample set, total RNA were prepared by and obtained from the Siteman Cancer Center Tissue Procurement Facility at Washington University (WU)-St. Louis from 16 CA and 15 AA colon cancer patients who underwent colon cancer surgery at Barnes Jewish Hospital. Here, total RNA was extracted from 31 pairs of snap-frozen tumors and adjacent matching normal colon using TRIzol followed by lithium precipitation (Invitrogen, Carlsbad, CA) according to the manufacturer’s protocol. For all samples, RNA quantity and quality was determined using a Nanodrop 2000C (Thermo Scientific, Waltham, MA).

### Agilent miRNA microarray analysis

Whole genome miRNA expression profiles for predicting colon cancer risk with respect to race were acquired using single color Agilent miRNA microarrays, release 16.0G4870A 6×60 K (Agilent Technologies, Foster City, CA). miRNAs were labeled and hybridized using Agilent miRNA Complete Labeling and Hybridization Kit (Agilent Technologies) in the Stony Brook University Genomics Core Facility. Labeled miRNA was used to probe and hybridize with Agilent Human Microarray Kit V3, 8×15 K arrays, based on Sanger miRbase release 12.0 (G4470C, Agilent Technologies). Hybridization was performed at 55°C for 20 h in a hybridization oven (Agilent Technologies) according to the manufacturer’s protocol. Hybridized microarrays were scanned with a DNA microarray scanner (Agilent G2565BA), and features were extracted using the Agilent Feature Extraction (AFE) image analysis tool (version A.10.7.3.1) with default protocols and settings. To ensure reliable results as to upregulation and downregulation in the miRNAs of a given patient, paired tumor/normal matched samples were run on the same array. All microarray data have been deposited in the NCBI GEO database with accession number GSE48267.

### Microarray preprocessing

The raw probe-level data were imported into GeneSpring 12.5GX (Agilent Technologies), and the miRNA signal was normalized using the default setting: threshold raw signal to 1.0, percent shift to 90th percentile as the normalization algorithm, and no baseline transformation. After normalization, the probes were filtered using the gIsGeneDetected flag provided in Agilent AFE software to remove miRNAs that were not detected in 60% of tumor or 60% of adjacent normal colon samples. Two class analysis of tumor vs. adjacent normal tissue RNA, AA tumor vs. CA tumor and AA adjacent normal vs. CA adjacent normal were conducted using Significance Analysis of Microarray (26) with a 1.5-fold change and FDR <0.05 for significance.

### qRT-PCR

For all samples, cDNA was synthesized from 100 ng of total RNA using the Universal cDNA Synthesis Kit (Exiqon, Woburn, MA). After cDNA conversion, quantification of candidate miRNAs (miR-182 and miR204) was determined using commercial specific microRNA LNA PCR primers (Exiqon). Real-time PCR was conducted using Universal RT SYBR Green master mix (Exiqon) in a Realplex real-time PCR machine (Eppendorf, Hauppauge, NY). Quantitative PCR was done under the following cycling conditions: 95°C for 10 min, and 45 cycles of 95°C for 10 s, 60°C for 1 min. All PCRs were done in triplicate. Transcript normalization of samples was obtained using miR-191 as a reference (27). The relative expression of the candidate miRNAs was measured by the threshold cycle Ct miR-191-Ct candidate miRNA. The effects of race, tumor, institutional source and first order interactions were analyzed by RANOVA (see below).

### Immunohistochemistry

The SBU FFPE tissue sections were deparaffinized and rehydrated according to standard protocols. Tissue sections were pretreated for antigen retrieval by heating in a microwave oven for 15 min in a 0.01 M citric acid solution (pH 6.0). Staining of FFPE colon specimens was performed according to the instructions for rapid immunohistochemical staining using Histostain-Plus Bulk Kit (Life Technologies, Grand Island, NY). Primary antibodies to FOXO1 (1:100 dilution/Cell Signaling Technology Inc., Davers, MA) and FOXO3 (1:250/Cell Signaling Technology Inc.) were used to detect these targets of miR-182 and miR-183. An isotypic antibody control was included with each tissue section.

### Scoring

A pathologist (N.O.), blinded to sample identity, evaluated the SBU tissue sections. The ‘intensity of staining’ of each tissue sample was scored using the following scale: 0, no staining; 1, light staining; 2, medium staining; and 3, maximal staining. Bootstrap RANOVA was used to determine significant differences of FOXO1 and FOXO3a expression by immunohistochemistry.

### Statistical analysis

Repeated measures analysis of variance (RANOVA) was implemented to evaluate significant effects in the model, with tumor as a within-subjects effect and race and institutional source of the RNA samples as between-subjects effects. Our analysis was based on a mixed model with individual effect on miRNA expression profile as a random effect, and tumor, race and data source as fixed effects: Y_hijk_ = μ + (Race_h_ + Tumor_j_ + Source_k_)^2^ + Sub_i(kh)_ + e_hijk_.

Here Y_hijk_ is the miRNA expression level for subject i, of race indicator h (h=1 or 0 if the subject is AA or CA, respectively), tumor indicator j (j=1 or 0 for tumor or normal tissue, respectively), and source indicator k (k=1 or 0 for SBU or WU, respectively). On the right hand side of the equation, μ is the overall mean, followed by the race, tumor, source individual effects, two-way and three-way interactions, the random subject effect Sub_i(kh)_, and the random error e_hijk_ independent of Sub_i(kh)_. Constraints ∑_h_Race_h_ = ∑_j_Tumor_j_ = ∑_k_Source_k_ = 0 are imposed for identifiability.

Since normality assumptions did not hold for these datasets, a non-parametric bootstrap approach was adopted to estimate the p-values (28). To further enhance the robustness of the tests, we considered both the p-values and the fold change to test for significance. The threshold was set as fold change >1.5 and p<0.05 for main effects. For interaction terms, the fold change threshold was set at 1.5 for either the AA tumor/CA tumor or the AA normal/CA normal ratio and the p-value threshold was set at 0.05.

## Results

### The effect of race and tumor on miRNA expression

Our strategy for selecting candidate miRNAs that could potentially be associated with biological differences between AA and CA colon cancers was to analyze the effect of race and tumor using RANOVA (see Materials and methods). Since the samples obtained from SBU and WU included differences in i) geographic location of the subjects; ii) processing and storage of the tissues (FFPE vs. snap frozen); and iii) RNA isolation procedure, the effect of institutional source was also included. While SAM (26) is extremely robust for two class comparisons, the advantage of RANOVA is that all three terms and first order interactions could be incorporated in the model.

The effect of the tumor was large compared to the effects of race or institutional source. RANOVA identified 49 miRNAs that were upregulated and 40 miRNAs that were downregulated in tumor vs. adjacent normal colonic tissue (Table I). This included the upregulation of a number of miRNAs (e.g., miR-21, miR-31, miR-96 and miR-135b, miR-182, miR-183) (14–19,29–34) and downregulation of miRNAs (e.g., 133a and mir-1) (31) which were previously noted.

As shown in Table II, 4 miRNAs demonstrated significant race:tumor interaction and 5 miRNAs showed both race and tumor main effects. Of the 5 miRNA with both race and tumor main effects, we noted the prominence of miR-182 and miR-204, which were identified based on RANOVA analysis of the SBU miRNA profiles alone (data not shown) and confirmed by the combined data sets. miR-182 was upregulated 11.1-fold in paired tumor vs. normal tissue and was increased 2-fold in AA vs. CA tumors. miR-204 was downregulated 3.6-fold in tumor vs. adjacent normal colonic tissue and increased 2.6-fold in AA vs. CA tumors.

### miR-182 demonstrates a significant race:tumor interactions based on qRT-PCR results

**Figure 1:**
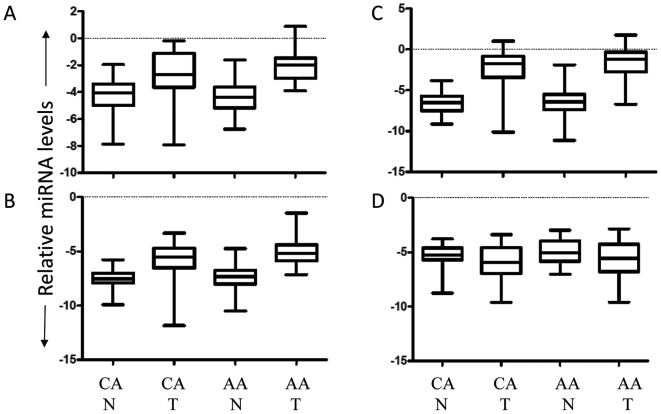
Confirmation by qRT-PCR analysis of miRNAs that were most differentially expressed. qRT-PCR analysis using primers for 25 of the most differentially expressed miRNAs were evaluated. Statistical significance was determined by RANOVA (p<0.05). Significance was as follows: (A) miR-182, CAN vs. AAT, CAT vs. AAT and AAN vs. AAT. (B) miR-183, CAN vs. AAT, CAT vs. AAT and AAT vs. AAN. (C) miR-135b, CAN vs. CAT and AAN vs. AAT. (D) miR-204, CAN vs. CAT and AAN vs. AAT. Abbreviations: CA, Caucasian American; AA, African American; T, tumor; and N, normal adjacent.

qRT-PCR using primers for 25 of the miRNAs which were most differentially expressed was conducted to confirm results of the miRNA microarray analysis. The miRNAs which exhibited statistical significance as determined by RANOVA analysis were mir-182 and miR-183 (Fig. 1). Other miRNAs (i.e., miR-135b and miR-204) exhibited significant difference between tumor and normal tissues only. Although the increased expression, as determined by miRNA microarray, of miR-204 in AA tumors compared to CA tumors could not be confirmed by qRT-PCR, our qRT-PCR analysis did confirm that of previous reports indicating downregulation of miR-204 in tumor vs. normal tissues.

**Figure 2:**
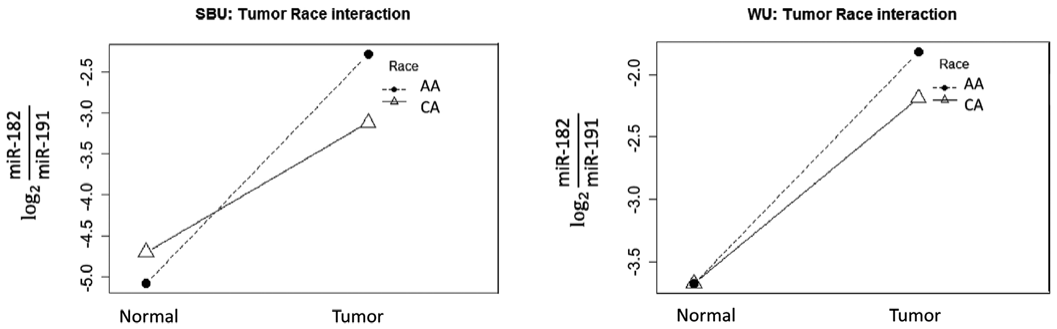
Three-way interactions between race (AA vs. CA) tumor (tumor vs. normal), and source (SBU vs. WU) for the miR-182 qRT-PCR data. Patients were classified into 8 groups (each group is determined by a combination of race, tumor and source) for their miR-182 relative expression level as measured by RT-PCR. For each data source, 4 associated group means were plotted in one figure, with a dot and triangle indicating race and an x-axis indicating normal and tumor. Additionally, the change in group means of CA patients between normal and tumor is signified by a solid line, while a dashed line represents this change in AA patients.

RANOVA analysis of only miR-182 and its cluster partner, miR-183 demonstrated a significant race:tumor interaction term (p<0.05, Table III). As demonstrated in Fig. 2, the lines connecting the paired tumor and normal tissues cross, thus graphically demonstrating the interaction between race and tumor in miR-182 qRT-PCR data. The relative level of miR-182 is much higher in the tumor than in the paired adjacent normal colonic tissue. In addition, the relative level of miR-182 is also consistently higher in AA tumors compared to CA tumors in both the SBU and WU cohorts singularly and in combination. Interestingly, there is little or no difference in AA normal mucosa compared to the CA normal tissue.

### Immunohistochemical analysis of potential miRNA targets

**Figure 3:**
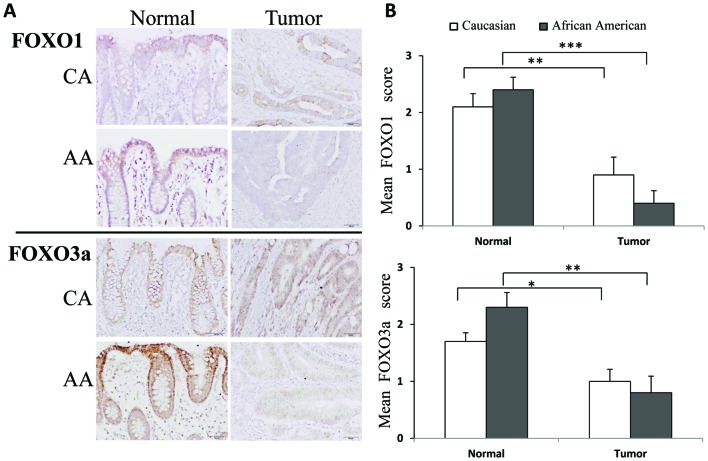
FOXO1 and FOXO3a staining in colon tumor and adjacent normal tissues. Immunohistochemical analysis of colon tissue from patients with colon cancer. Representative images (magnification, ×400) of immunohistochemically stained tissues from patients of CA and AA origin are shown along with a corresponding graph depicting the intensity of staining. Vertical bars, standard error of the mean; n=10 for each group. The intensity of the staining was scored using the scale: 0, no staining; 1, low staining; 2, medium staining; and 3, strong staining. ^*^p<0.05, ^**^p<0.005, and ^***^p<0.001.

For further downstream analysis, we focused our attention on the potential targets of miR-182 (35,36). We reasoned that upregulation of miR-182 could result in reduced expression of target mRNAs. To test this hypothesis, immunohistochemical staining for two potential miR-182 targets, FOXO1 and FOXO3a, were performed on 10 of the 15 matching tissue sets from SBU CA and AA colon cancer patients (Fig. 3). SBU samples were solely used due to availability of samples for this analysis. FOXO1 and FOXO3a cytoplasmic staining was detected in all normal colon tissue samples regardless of race. In normal colonic mucosa, staining for both FOXO1 and FOXO3a was most intense in luminal surface columnar cells, but colonic crypt epithelial cells also showed weak to moderate cytoplasmic staining. In tumors, FOXO1 was localized exclusively in the cytoplasm, but FOXO3a showed nuclear staining, potentially reflecting the translocation of phospho-FOXO3a. The expression of FOXO1 decreased by 57.14% in tumors of CAs and decreased by 83.33% in tumors of AAs as compared to adjacent normal tissues. Similarly, the extent of expression of FOXO3a decreased by 41.17% in tumors of CAs and decreased by 65.22% in tumors of AAs as compared to adjacent normal tissues. However, the trend towards reduced staining in AA tumors compared to CA did not reach statistical significance. Statistical significance was noted only when assessing differences in staining between normal and tumor tissues.

## Discussion

This exploratory study provides proof of principle for identifying biological factors that could contribute to increased mortality in CRC in AAs compared to CAs. Whole human genome miRNA expression profiles were compared between AA and CA paired colon cancer and adjacent normal colonic tissue. To increase the power of the analysis, samples were combined between two medical centers located, respectively, in New York City, NY and St. Louis, MO, with at least 30 subjects per each racial cohort. The 2 cohorts were not significantly different with respect to distribution of age or gender. However, there were significant differences between the 2 cohorts with respect to the type of samples used: formalin fixed paraffin embedded tissues vs. snap frozen tissues.

One approach towards identifying candidate miRNAs could be to conduct a series of two class comparisons using SAM (26); i) compare paired tumor vs. adjacent normal; ii) compare unpaired AA tumor with CA tumor; and iii) compare unpaired AA normal with CA normal. The miRNAs that were differentially expressed in tumor vs. normal and between AA cancer and CA cancer would be selected as potential candidates. However, to simultaneously evaluate the effect of race, tumor and the institutional source as well as first order interaction terms we instead used RANOVA.

The largest differences in miRNA expression are between tumor and adjacent normal tissue. RANOVA identified miRNAs that were previously reported to be differentially expressed between tumor and normal colon tissue (14–19,29–34). RANOVA identified nine miRNAs that exhibited either a race:tumor interaction or both race and tumor main effects. We focused on one miRNA that were initially identified by both SAM and RANOVA analysis in the SBU cohort and then confirmed in the analysis of the combined SBU and WU cohorts. Our main potential biomarker for indicating a role in racial disparity, miR-182, has been previously reported to be upregulated in tumor vs. normal tissues. In addition, miR-204, which has been previously reported to be downregulated in tumors (29–34) was also considered as a potential biomarker. While subsequent qRT-PCR confirmed upregulation of miR-182 and downregulation of miR-204 in tumor vs. normal tissues, only miR-182 was confirmed as being increased in AA vs. CA tumor tissues. In agreement, analysis of miR-182 expression in the 6 AA designated colon cancers and the 37 CA designated colon cancers in The Cancer Genome Atlas database also showed increased expression in the AA cancers, but this increase did not reach statistical significance (37). This could be due in part to the small sample number of AA colon cancer tissue. A recent RT-PCR analysis of selected miRNAs in AA and CA paired tumor and adjacent normal colon tissues detected an effect of race and colon cancer stage on expression; however, miR-182 was not measured in this study (38). While the observation that miR-182 expression is increased in AA tumors, needs to be confirmed in larger samples sets and/or better annotation of racial metadata in existing collections, the results of this study suggests that variations in sample processing at different institutions will not obscure racial differences in miRNA expression.

In accordance with the expression levels of miR-182, there were reduced expressions of FOXO3 and FOXO1 in tumor vs. normal tissues in the SBU cohort. Although expression levels were noted to differ between AA vs. CA tumors, this decreased expression was not statistically significant. These immunohistochemical results support a recent report of decreased FOXO3a (39) expression in CRC, and suggest that miR-182 levels may contribute to the regulation of FOXO1 and FOXO3a expression. The concept that increased miR-182 levels in AA compared to CA levels may contribute to increased AA colon cancer mortality is further supported by a recent report linking miR-182 to reduced colon cancer survival and increased liver metastases (40). Targeted suppression of miR-182 expression has been reported to reduce hepatic metastases in an experimental model of melanoma (41), suggesting that miR-182 is involved in promoting the development of liver metastases.

The molecular pathways leading AA colon cancer racial health disparity remain to be determined. We propose that this may be related to an increase of miR-182 in AA colon cancers. One potential link may be with defective mismatch repair (19). AAs with CRC (43%) have a higher proportion of MSI instability compared to the general US population (42). This instability may in turn be associated with altered prognosis and response to chemotherapeutic agents (42). Another may be epigenetic modulation of the miR-182 locus (43). Identification of the biological pathways associated with differential expression of miRNAs in AA cancers will, therefore, require the generation and integration of parallel genetic and epigenetic mRNA expression datasets with an expanded racially annotated miRNA colon cancer dataset.

## Figures and Tables

**Table I:** miRNAs that were significantly upregulated and downregulated in tumor vs. paired adjacent normal colon tissues.

Upregulated miRNA	Fold change tumor/normal	p-value	Downregulated miRNA	Fold change tumor/normal	p-value
hsa-miR-135b	46.25	0	hsa-miR-133a	0.04	0
hsa-miR-183	20.00	0	hsa-miR-139-5p	0.07	0
hsa-miR-31	17.05	0	hsa-miR-378^*^	0.09	0
hsa-miR-96	11.95	0	hsa-miR-133b	0.10	0
hsa-miR-182	11.12	0	hsa-miR-1	0.10	0
hsa-miR-552	8.60	0	hsa-miR-30a^*^	0.11	0
hsa-miR-224	5.47	0	hsa-miR-490-3p	0.13	0
hsa-miR-503	4.81	0	hsa-miR-149	0.14	0
hsa-miR-18a	4.43	0	hsa-miR-363	0.16	0
hsa-miR-17^*^	3.90	0	hsa-miR-145^*^	0.18	0
hsa-miR-409-3p	3.65	0	hsa-miR-129-3p	0.18	0
hsa-miR-424	3.49	0	hsa-miR-143^*^	0.21	0
hsa-miR-203	3.42	0	hsa-miR-145	0.23	0
hsa-miR-18b	3.38	0.001	hsa-miR-218	0.23	0
hsa-miR-7	3.36	0	hsa-miR-204	0.28	0.004
hsa-miR-21^*^	3.24	0	hsa-miR-143	0.29	0
hsa-miR-629^*^	3.15	0	hsa-miR-23b^*^	0.29	0
hsa-miR-19a	2.98	0.001	hsa-miR-29c^*^	0.31	0
hsa-miR-424^*^	2.91	0	hsa-miR-338-3p	0.33	0
hsa-miR-21	2.80	0	hsa-miR-195	0.35	0
hsa-miR-181c	2.67	0.002	hsa-miR-24-1^*^	0.41	0.002
hsa-miR-34b^*^	2.58	0	hsa-miR-30a	0.41	0
hsa-miR-148a	2.48	0.003	hsa-miR-662	0.42	0.003
hsa-miR-34a	2.28	0.02	hsa-miR-497	0.42	0
hsa-miR-221	2.27	0.001	hsa-miR-99a	0.44	0
hsa-miR-146a	2.18	0	hsa-miR-451	0.46	0
hsa-miR-301a	2.16	0.002	hsa-miR-28-3p	0.48	0
hsa-miR-501-3p	2.16	0	hsa-miR-30e^*^	0.49	0.01
hsa-miR-1246	2.10	0.003	hsa-miR-378	0.50	0
hsa-miR-29b	2.09	0.004	hsa-miR-125b-2^*^	0.50	0.002
hsa-miR-130b	2.05	0	hsa-miR-149-2^*^	0.51	0.006
hsa-miR-663b	2.04	0.001	hsa-miR-610	0.52	0.015
hsa-miR-20a	2.03	0	hsa-miR-30c-1^*^	0.53	0.005
hsa-miR-296-5p	2.03	0	hsa-miR-100	0.56	0
hsa-miR-17	1.95	0	hsa-miR-30c	0.57	0
hsa-miR-92a	1.89	0	hsa-miR-193b^*^	0.59	0.044
hsa-miR-429	1.85	0.007	hsa-miR-28-5p	0.62	0.006
hsa-miR-19b	1.83	0	hsa-miR-125b	0.62	0.002
hsa-miR-335	1.78	0.018	hsa-miR-365	0.66	0
hsa-miR-1249	1.75	0.044	hsa-let-7e	0.66	0.002
hsa-miR-141	1.73	0.035			
hsa-miR-29a	1.68	0			
hsa-miR-142-3p	1.66	0.016			
hsa-miR-93	1.66	0			
hsa-miR-663	1.65	0.002			
hsa-miR-148b	1.59	0.033			
hsa-miR-425	1.58	0.046			
hsa-miR-25	1.57	0			
hsa-miR-1280	1.51	0			

RANOVA was used to identify miRNAs that were differentially expressed (1.5-fold, p<0.05).

**Table II:** miRNAs with significant race:tumor interaction or both significant race and tumor main effects.

	Race:tumor	Race	Tumor	Source	Source:race	Source:tumor	Source:race:tumor
miR-H1	**0.016**	0.225	0.001	0.16	0.099	0	0.033
miR-210	**0.022**	0.226	0.001	0.074	0.441	0.297	0.049
miR-128	**0.036**	0.754	0.013	0.001	0.491	0.8	0.555
miR-200a	**0.048**	0.452	0.022	0.278	0.974	0.905	0.262
miR-152	0.57	**0.012**	**0.014**	0.013	0.671	0.323	0.444
miR -204	0.948	**0.013**	**0.004**	0.239	0.288	0.841	0.325
miR-182	0.959	**0.015**	**0**	0.569	0.074	0.809	0.856
miR-222	0.665	**0.017**	**0**	0	0.332	0.065	0.196
miR-202	0.164	**0.045**	**0.017**	0	0.018	0.021	0.661

The p-values for the effect of race, tumor, institutional source of the tissue and all first order interactions were determined using RANOVA (see Materials and methods). The threshold for significance was set as p<0.05.

**Table III:** RANOVA analysis of qRT-PCR analysis of selected miRNA expression relative to miR-191.

	Race:tumor	Race	Tumor	Source	Source:race	Source:tumor	Source:race:tumor
hsa-miR-182	**0.001**	0.325	0	0	0.905	0.058	0.266
hsa-miR-204	0.439	0.898	**0**	0.038	0.028	0.053	0.788
hsa-miR-183	**0.048**	0.048	0	0.473	0.473	0.189	0.943
